# The Comparative Energy of Nutrients

**Published:** 1887-08

**Authors:** 


### THE COMPARATIVE ENERGY OF NUTRIENTS.

It is a philosophical fact, conceded upon all sides, that the sun is the parent of all the physical forces which display themselves upon the face of the earth, and that the various forms of matter are capable of exerting a force by combustion, or other chemical change, in the exact ratio of the quantity of solar heat absorbed in their growth or formation.

It was just fifty years ago that Dr. Fr. Mohr, a young German chemist, advanced the theory that apart from the chemical elements, "there is in the physical world one agent only, and this is called energy. It may appear, according to circumstances, as motion, chemical affinity, cohesion, electricity, light, magnetism, and from any one of these forms it can be be transferred into any of the others."

In view of the well known conservatism of the medical faculty, it is not surprising that the essay of Dr. Mohr, then so much in advance of accepted theories, was refused by the leading journal of physical science of his own country, although subsequently published in the Austrian "Zeitschrift fur Physik und verwandte Wissenschraften," at Vienna. Since this period, however, the attention of scientists has been more searchingly directed to a subject of such general importance, and the announcement of Dr. Mohr has passed into a philosophic aphorism. Thus is it that many of the more essential truths affecting life and health have had to fight their way to recognition.

The energy exerted by men and animals, not alone physical, but mental, of whatever shade or degree, is derived from that great reservoir of force, the sun.

Entering the system in the form of nutrients of various kinds, all the life giving qualities they possess may be traced through all the changes and gradations, to which they have been subject, to this common origin. In order to test the relative value of those life-giving substances, which nature has so bountifully supplied and is constantly supplying, a very delicate apparatus has been devised for measuring the degrees of heat separately evolved from them in the process of consumption, which is denominated the "Colorimeter."

From experiments thus made by eminent chemists, the potential energy of the various articles of food common to civilized life, have been tabulated with a certain degree of accuracy. It is from such estimates that the following table has been prepared, the maximum being stated at one hundred, and the several articles relieved of all refuse matter:

| | |
|---|---|
| Butter | 100 |
| Oleomargarine | 99 |
| Pork, very fat | 93 |
| Beef (flank), very fat | 74 |
| Cheese, whole milk | 55 |
| Smoked ham | 53 |
| Mutton (side), well fattened | 51 |
| Oatmeal | 49 |
| Sugar | 48 |
| Mutton, loin (chops) | 47 |
| Wheat flour | 44 |
| Rice | 44 |
| Corn (maize), meal | 43 |
| Rye flour | 43 |

| | |
|---|---|
| Beans | 41 |
| Peas | 39 |
| Beef (side), well fattened | 39 |
| Salt mackerel | 37 |
| Smoked herring | 36 |
| Mutton (shoulder) | 35 |
| Wheat bread | 34 |
| Beef (sirloin), rather fat | 31 |
| Cheese, skimmed milk | 31 |
| Mutton (leg) | 31 |
| Beef (neck) | 30 |
| Canned salmon | 27 |
| Mackerel, very fat | 26 |
| Salmon | 26 |
| Beef (round), rather lean | 21 |
| Hen's eggs | 20 |
| Shad | 20 |
| Mackerel, average | 18 |
| Mackerel, rather lean | 12 |
| Potatoes | 11 |
| Salt cod | 11 |
| Sweet potatoes | 11 |
| Blue fish | 10 |
| Haddock | 9 |
| Cod | 8 |
| Cow's milk | 8 |
| Flounder | 7 |
| Oysters | 6 |
| Cow's milk, skimmed | 4 |
| Turnips | 3 |

